# Anaplastic ganglioglioma in pregnancy a cause of cerebral edema and maternal death

**DOI:** 10.1515/crpm-2022-0002

**Published:** 2022-04-07

**Authors:** Luisa F. Capera, Rafael L. Aragón Mendoza, Roberto Gallo Roa, Viviana Dávila Romero

**Affiliations:** Obstetrics and Gynecology Resident, Universidad De La Sabana, Hospital Universitario de La Samaritana, Bogotá, Colombia; Specialist in Maternal-Fetal Medicine, Hospital Universitario De La Samaritana, Bogotá, Colombia; Obstetrics and Gynecology Specialist, Hospital Universitario De La Samaritana, Bogotá, Colombia

**Keywords:** brain tumor, ganglioglioma, pregnancy

## Abstract

**Objectives:**

The true incidence of anaplastic ganglioglioma during pregnancy is extremely rare, very few cases have been reported in the literature.

**Case presentation:**

This is a report of a case of anaplastic ganglioglioma diagnosed in pregnancy. The patient is a 23-year-old primigravida who presented at 19 weeks of gestation headache and a convulsive episode. Her workup revealed a rare cerebral tumor that progressed to a neurological decline and died during the postpartum period.

**Conclusions:**

Anaplastic ganglioglioma is an aggressive counterpart of Glial tumors; in pregnancy they are rare and symptoms are nonspecific. The outcome for the mother in this case fatal and a protocol for these cases has not yet been reported.

## Introduction

The incidence of anaplastic ganglioglioma during pregnancy is unknown, very few cases have been reported. Its diagnosis is rarely achieved in a timely manner, since the clinical presentation may be non-specific and instead taken for physiologic pregnancy-related symptoms [1, 2]. As such, poor outcomes for both mother and fetus have been described. We present the case of a 19-weeks gravid, 23-year-old patient, with a past medical history remarkable for neurofibromatosis presenting with headache alongside a first convulsive episode. Cerebral magnetic resonance imaging was performed, revealing a pineal region tumor and non-communicating hydrocephalus requiring partial resection and ventriculo-atrial shunt surgery. Biopsies were taken, and a histopathologic diagnosis of anaplastic ganglioglioma was made. Our patient did not accept adjuvant treatment, and was diagnosed with severe preeclampsia remote from term. Urgent delivery through cesarean section yielded a live birth. The patient progressed to neurologic impairment and cerebral edema leading to cardiovascular arrest and post-partum death.

## Case presentation

A 23 year-old primigravida, 19 weeks pregnant, with a past medical history remarkable for neurofibromatosis type 1; was admitted for 15 days of non-localized headache with a pain intensity score of 6/10, alongside a first convulsive episode consisting on a generalized tonic-clonic seizure. On admission, vital signs were within normal limits. On neurologic assessment, the patient had a Glasgow Coma Score of 15, but bradylalia, neck stiffness and bilateral papilledema were found on examination.

**Figure 1:**
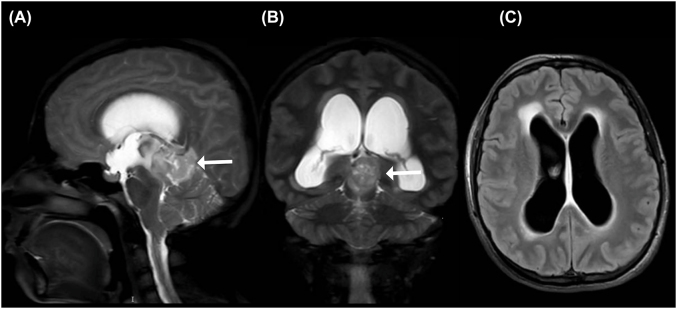
Brain magnetic resonance imaging. (A, B) Mass in the pineal projection area with a heterogeneous signal with areas of intralesional necrosis (white arrow), obliteration of the supra and infratentorial subarachnoid space with a compressive effect on the midbrain, bridge and medulla. (C) Changes suggestive of ventriculomegaly, dilation of the supratentorial ventricular system with transependymal migration.

The patient was hospitalized for neurologic monitoring and levetiracetam was administered for the control of seizures. Fetal well-being was confirmed through obstetric ultrasonography and a brain magnetic resonance imaging (MRI) was performed, revealing a 43 mm mass projecting to the pineal region. The mass displayed heterogeneous enhancement due to areas of necrosis, and exerted a compressive effect on the midbrain, pons and brainstem. The latter lead to obstructive hydrocephalus as a result of the herniation of cerebellar tonsils through the foramen magnum (Figure 1). The patient was taken to partial mass resection and endoscopic ventriculostomy. A hypervascularized lesion was found on the posterior wall of the third ventricle. Biopsies were taken, and histopathologic studies revealed a high-grade anaplastic ganglioglioma (WHO grade III), with 40% Ki-67 immunostaining. After surgery, the patient had an uneventful recovery and was discharged on levetiracetam for out-patient management.

A multidisciplinary team meeting was held with the participation of specialists in gynecology, maternal-fetal medicine, neurology, neurosurgery, oncology, psychiatry, psychology and medical ethics. Consensus was reached on offering the patient to be treated with chemotherapy and radiation therapy during pregnancy. Risks and benefits for both mother and fetus were discussed with the patient, she decided against receiving treatment.

**Figure 2:**
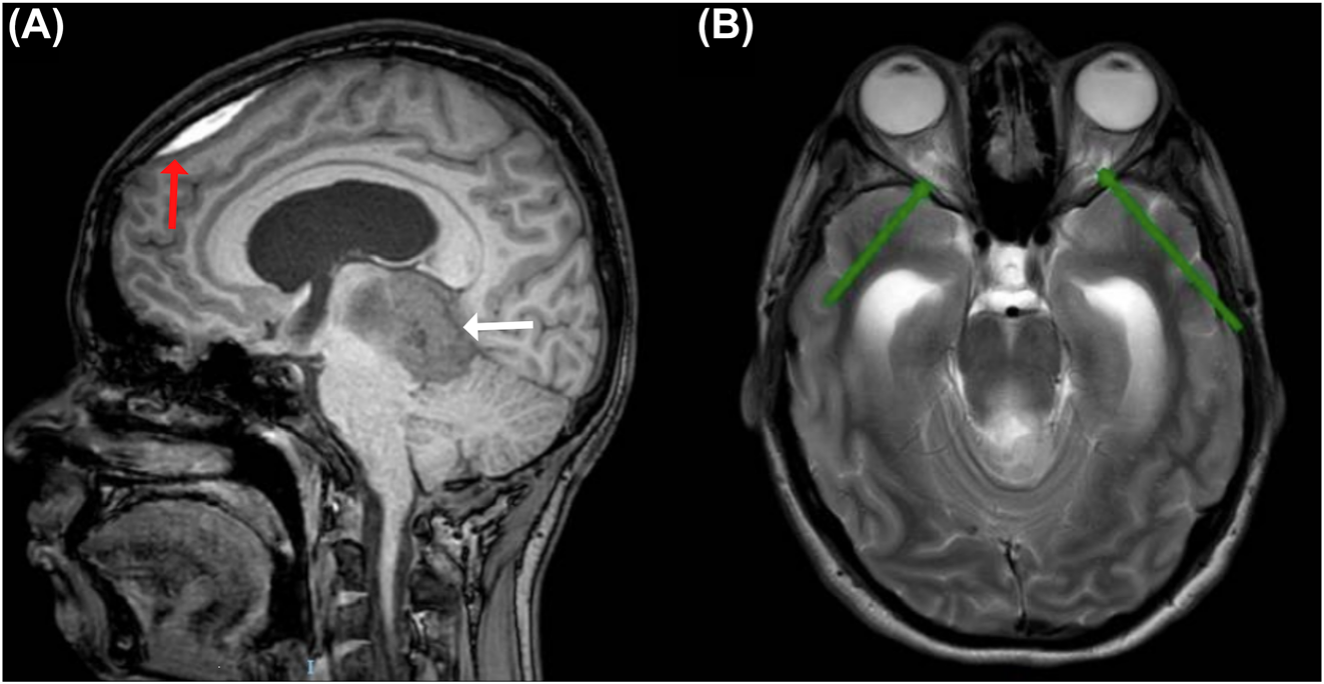
Follow-up brain magnetic resonance imaging. (A) Subdural hematoma adjacent to the frontal bone of 43 mm, with minimal compressive effect on the adjacent frontal parenchyma (red arrow), mass in the pineal region corresponds to ganglioglioma (white arrow) (B) Increase in cerebrospinal fluid around the optic nerves with a tortuous course in the intraconal segment and protrusion of the optic discs (green arrow).

**Figure 3:**
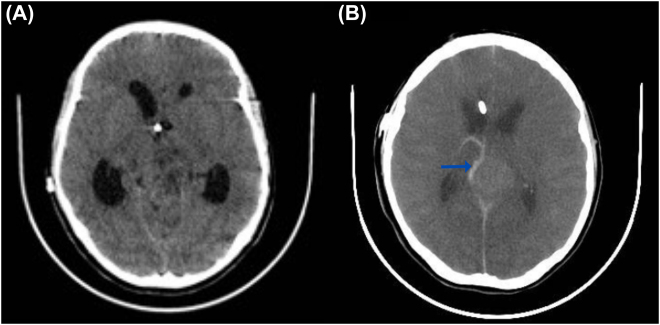
Brain computed tomography. (A) Loss of differentiation of the sulci and white matter with gray matter due to cerebral edema. (B) Loss of differentiation between the basal ganglia and the white matter; adjacent to mass, ganglioglioma, area of hemorrhage is observed (blue arrow).

She continued out-patient treatment, and at pregnancy week 22, our patient had to be admitted due to seizures. The dose of levetiracetam was adjusted and a new brain MRI was performed revealing supratentorial ventriculomegaly due to chronic hydrocephalus, a subdural hematoma adjacent to the frontal bone, and increased cerebrospinal fluid surrounding the optic nerves (Figure 2). Symptoms of intracranial hypertension were persistent. Therefore, ventriculoatrial shunting was conducted and the patient was discharged. At 30 weeks of pregnancy, she presented to the emergency room for a new episode of non-localized headache and seizures. On admission, blood pressure was high, liver function tests became anormal and a diagnosis of severe preeclampsia was made. Urgent delivery through cesarean section yielded a live birth (1,340 g, APGAR 6/8/9 on minutes 1, 5 and 10, respectively). During the immediate puerperium, her neurologic status deteriorated progressively, leading to ventilatory support requirement. A head computed tomography (CT) was performed, finding increased hydrocephalus and cerebral edema (Figure 3). On postoperative day 8, brain death was diagnosed.

## Discussion

The Ganglioglioma is a rare tumor, representing less than 2% of all brain tumors [3]. This tumor is classified as a mixed neoplasm of the central nervous system (CNS), composed by neoplastic glial cells and dysplastic neurons [3] according to the 2016 World Health Organization (WHO) classification of tumors of the CNS. Grade III or Anaplastic gangliogliomas represent 1–5% of all gangliogliomas, which display increased cellularity, nuclear atypia and increased mitotic activity [4]. These tumors may present as “de novo” cases or malignant transformation of benign gangliogliomas [5]; Very few cases have been reported during pregnancy, and even less regarding the anaplastic subtype [1], [2], [3].

The clinical manifestations may be non-specific and instead taken for physiologic pregnancy-related symptoms [6]. Headache is the most common symptom, and has been reported in 90% of all cases [1], seizures have been described in 40% of patients [7] and specific characteristics may appear depending on the origin. For instance, visual symptoms may appear when the occipital cortex is affected [6]. The increase of symptoms may be at least partly explained by the physiologic hemodynamic changes occurring during pregnancy. For example, the intravascular volume increases, leading to a greater blood flow towards the tumor and fluid extravasation towards the extracellular space, generating peritumoral edema [4]. Also, in up to 75% of cases, tumor growth may occur [7] due to the production of growth factors such as the placental growth factor, insulin-like growth factor and vascular endothelial growth factor, all of which promote tumor angiogenesis [8].

When suspecting brain tumors during pregnancy, a brain MRI is the imaging of choice since it does not employ ionising radiation [1]. The ganglioglioma lacks specific characteristics on imaging. Most gangliogliomas are poorly defined solid or solid-cystic masses with irregular margins, which contrast enhancement. They may present as iso or hyposignal on T1-weighted images, and hypersignal on T2-weighted images [4, 9]. The location is variable, but are most frequently found in the temporal and frontal regions [5].

Treatment for this condition is the same during pregnancy than in general population, and consists in surgical resection aiming to improve symptoms, survival and the obtention of a histopathologic diagnosis. Maternal-fetal outcomes are not worsened by surgical treatment [10] and there are greater survival rates when complete tumor resection is achieved [11].

Anaplastic ganglioglioma has an aggressive behavior. As such, better control of disease has been reported when local radiation and adjuvant chemotherapy are used in addition to surgery [11]. Chemotherapy during the first trimester of pregnancy has been associated with spontaneous miscarriage, CNS malformations and hematopoietic disturbances. During the second and third trimester, chemotherapy is relatively safe. However, some of the reported risks include pre-term labor, low birthweight, and fetal myelosuppression. Therefore, it is recommended to stop chemotherapy 3 weeks before the estimated due date [10, 12]. Radiotherapy has been associated with teratogenesis, intrauterine growth restriction, cognitive developmental disorder and childhood malignancy. The risk increases if radiotherapy is delivered during organogenesis or when the fetus radiation dose exceeds 100 mGy, which is rarely the case for therapeutic radiotherapy [10]. Fetal radiation exposure may be minimized by modifying the imaging area and the beam size to more than 30 cm, as well as wearing a 1 cm thick lead apron for abdomen protection, among other strategies [12, 13]. To the best of our knowledge, no cases of anaplastic ganglioglioma treated with chemotherapy or radiotherapy during pregnancy have been described to date [2]. Most cases during pregnancy report surgical excision, pre-term labor and posterior adjuvant therapy [1, 2]. Delays in treatment due to the risk of possible adverse fetal outcomes will negatively affect survival in these patients [8].

Pregnancy does not increase the risk of progression or transformation of brain tumors from lower to high grades [5]. However, brain tumors have been associated with worse maternal-fetal outcomes such as preterm birth, intrauterine growth restriction, hydrocephalus, maternal neurologic impairment and seizures leading to aspiration pneumonia [14]. Furthermore, higher rates of cesarean sections have been reported due to the theorical risk of increased intracranial pressure during labor, cerebral edema and increasing the risk of tumor hemorrhage [1, 10, 14].
